# Porphyria‐Safe Emergency Management in COVID‐Associated GBS‐Like Neuropathy: Why Early Medication Review Should Accompany PBG/ALA Testing

**DOI:** 10.1002/ccr3.73128

**Published:** 2026-07-07

**Authors:** Muhammad Abdullah Awan

**Affiliations:** ^1^ Wah Medical College National University of Medical Sciences Rawalpindi Pakistan

**Keywords:** acute intermittent porphyria, COVID‐19, Guillain‐Barre syndrome, medication safety, porphobilinogen, porphyrinogenic drugs

## Abstract

In COVID‐associated acute neuropathy with abdominal pain, seizures, hyponatremia, hypertension, or hepatic involvement, suspected acute intermittent porphyria should prompt both early PBG/ALA testing and immediate porphyria‐safe medication review to avoid worsening neurovisceral attacks before diagnostic confirmation.

Dear Editor,

I read with great interest the case report by Sadeghi et al., titled “Acute Intermittent Porphyria Triggered by COVID‐19 Mimicking Guillain‐Barre Syndrome: A Diagnostic Challenge,” published in Clinical Case Reports [1]. The authors describe a 16‐year‐old girl who developed persistent abdominal pain, seizures, hyponatremia compatible with SIADH, hypertension, transaminitis, and rapidly progressive acute motor neuropathy after COVID‐19 infection. The diagnostic pathway was complex because the presentation initially resembled Guillain‐Barre syndrome and multisystem inflammatory syndrome in children, before urine porphobilinogen positivity, weakly positive serum aminolevulinic acid, urine darkening, and a pathogenic HMBS variant confirmed acute intermittent porphyria [1].

The case is valuable because it reminds clinicians that acute intermittent porphyria can mimic post‐infectious inflammatory neuropathies. However, one practical point deserves stronger emphasis: in suspected porphyric neuropathy, diagnosis and emergency management should proceed together. Early PBG/ALA testing is essential, but it should be accompanied by immediate review of all prescribed drugs, especially antiepileptics, antibiotics, sedatives, antiemetics, hormonal agents, and other medications that may be unsafe in acute hepatic porphyria.

This distinction is clinically important in the reported case because phenytoin was used during urgent seizure management and was later replaced with levetiracetam after acute porphyria was suspected because of its porphyrinogenic potential [1]. We agree that emergency seizure control may sometimes require rapid treatment before the full metabolic diagnosis is clear. Nevertheless, when seizures occur together with unexplained abdominal pain, hyponatremia, hypertension, autonomic symptoms, transaminitis, and acute axonal motor neuropathy, the possibility of acute intermittent porphyria should trigger a porphyria‐safe medication checklist even before confirmatory genetic testing is available.

In similar cases, a simple emergency algorithm may reduce avoidable risk. First, clinicians should recognize a red‐flag cluster: severe or recurrent abdominal pain, neuropsychiatric symptoms, seizures, autonomic instability, hyponatremia, darkening urine, hepatic enzyme elevation, and motor‐predominant axonal neuropathy. Second, fresh urine PBG and ALA testing should be requested urgently, ideally with attention to sample protection and appropriate handling. Third, potentially porphyrinogenic medications should be stopped or substituted where clinically feasible. Fourth, seizure control should favor safer alternatives when available, while hypertension, electrolyte disturbance, pain, and nutritional status should be managed aggressively. Finally, access to hemin or other porphyria‐directed therapy should be explored early rather than after neurological deterioration.

The authors appropriately report that the patient improved after 10% dextrose infusion, a high‐carbohydrate diet, supportive management, and withdrawal of phenytoin, while hemin was unavailable through the national health‐care system [1]. This highlights another lesson for resource‐limited settings: when definitive therapy is delayed or inaccessible, avoiding harmful triggers becomes even more important. A porphyria‐safe medication review is low‐cost, rapidly implementable, and may prevent iatrogenic worsening while biochemical and genetic confirmation are pending.

A second reporting issue concerns how COVID‐19 is framed as the trigger. The temporal association is plausible, and the authors provide a strong clinical narrative [1]. Yet COVID‐19 may act not only as an infectious precipitant but also indirectly through reduced oral intake, physiologic stress, inflammatory activation, diagnostic anchoring toward GBS or MIS‐C, and exposure to medications used during acute care. Future case reports of infection‐associated AIP should therefore report not only the infectious timeline but also fasting or caloric restriction, medication exposures before and during admission, hormonal factors, and timing of symptom escalation after each intervention.

The electrodiagnostic discussion in the case is particularly useful because the repeat study favored acute asymmetric axonal motor neuropathy rather than a classic demyelinating GBS pattern [1]. We suggest that future reports pair this neurophysiologic distinction with an emergency safety statement: when a GBS‐like illness shows axonal motor predominance plus neurovisceral and autonomic features, clinicians should simultaneously test for porphyria and review medication safety. This dual approach prevents the diagnostic process from becoming separated from immediate risk reduction.

Overall, Sadeghi et al. present an educational and clinically important case that expands awareness of COVID‐19‐associated AIP presenting as a GBS‐like neuropathy [1]. Its broader message is that early recognition should not stop at ordering urine PBG and ALA. In acute neurovisceral presentations, especially in adolescents with seizures and abdominal pain, a porphyria‐safe medication review should become an emergency step alongside biochemical testing, nutritional support, and planning for porphyria‐directed therapy.

## 1 Conclusion

This case usefully demonstrates that COVID‐19 may precipitate acute intermittent porphyria and mimic Guillain‐Barre syndrome. However, similar future cases should emphasize that suspected AIP requires immediate porphyria‐safe medication review in parallel with PBG/ALA testing. Such an approach may reduce iatrogenic risk, especially when hemin access is delayed or unavailable.

## Author Contributions

**Muhammad Abdullah Awan:** conceptualization, formal analysis, project administration, writing – original draft, writing – review and editing.

## Funding

The author has nothing to report.

## Ethics Statement

Ethics approval was not required for this Letter to the Editor because it discusses a previously published case report and does not include new patient data.

## Consent

Patient consent was not required for this Letter to the Editor because no new identifiable patient information is presented.

## Conflicts of Interest

The author declares no conflicts of interest.

## Data Availability

No new data were generated or analyzed for this letter.
